# Characterization of the Microbial Resistome in Conventional and “Raised Without Antibiotics” Beef and Dairy Production Systems

**DOI:** 10.3389/fmicb.2019.01980

**Published:** 2019-09-04

**Authors:** Pablo Rovira, Tim McAllister, Steven M. Lakin, Shaun R. Cook, Enrique Doster, Noelle R. Noyes, Maggie D. Weinroth, Xiang Yang, Jennifer K. Parker, Christina Boucher, Calvin W. Booker, Dale R. Woerner, Keith E. Belk, Paul S. Morley

**Affiliations:** ^1^Department of Animal Sciences, College of Agricultural Sciences, Colorado State University, Fort Collins, CO, United States; ^2^Lethbridge Research and Development Centre, Agriculture and Agri-Food Canada, Lethbridge, AB, Canada; ^3^Department of Microbiology, Immunology and Pathology, College of Veterinary Medicine and Biomedical Sciences, Colorado State University, Fort Collins, CO, United States; ^4^Alberta Agriculture and Forestry, Lethbridge, AB, Canada; ^5^Veterinary Population Medicine Department, University of Minnesota, St. Paul, MN, United States; ^6^Department of Animal Sciences, University of California, Davis, Davis, CA, United States; ^7^Department of Molecular Biosciences, University of Texas, Austin, TX, United States; ^8^Department of Computer and Information Science and Engineering, University of Florida, Gainesville, FL, United States; ^9^Feedlot Health Management Services, Ltd., Okotoks, AB, Canada; ^10^Department of Animal and Food Sciences, College of Agricultural Sciences & Natural Resources, Texas Tech University, Lubbock, TX, United States; ^11^VERO - Veterinary Education, Research, and Outreach Program, Texas A&M University and West Texas A&M University, Canyon, TX, United States

**Keywords:** cattle, antibiotic resistance, resistome, microbiome, metagenomics

## Abstract

Metagenomic investigations have the potential to provide unprecedented insights into microbial ecologies, such as those relating to antimicrobial resistance (AMR). We characterized the microbial resistome in livestock operations raising cattle conventionally (CONV) or without antibiotic exposures (RWA) using shotgun metagenomics. Samples of feces, wastewater from catchment basins, and soil where wastewater was applied were collected from CONV and RWA feedlot and dairy farms. After DNA extraction and sequencing, shotgun metagenomic reads were aligned to reference databases for identification of bacteria (Kraken) and antibiotic resistance genes (ARGs) accessions (MEGARes). Differences in microbial resistomes were found across farms with different production practices (CONV vs. RWA), types of cattle (beef vs. dairy), and types of sample (feces vs. wastewater vs. soil). Feces had the greatest number of ARGs per sample (mean = 118 and 79 in CONV and RWA, respectively), with tetracycline efflux pumps, macrolide phosphotransferases, and aminoglycoside nucleotidyltransferases mechanisms of resistance more abundant in CONV than in RWA feces. Tetracycline and macrolide–lincosamide–streptogramin classes of resistance were more abundant in feedlot cattle than in dairy cow feces, whereas the β-lactam class was more abundant in dairy cow feces. Lack of congruence between ARGs and microbial communities (procrustes analysis) suggested that other factors (e.g., location of farms, cattle source, management practices, diet, horizontal ARGs transfer, and co-selection of resistance), in addition to antimicrobial use, could have impacted resistome profiles. For that reason, we could not establish a cause–effect relationship between antimicrobial use and AMR, although ARGs in feces and effluents were associated with drug classes used to treat animals according to farms’ records (tetracyclines and macrolides in feedlots, β-lactams in dairies), whereas ARGs in soil were dominated by multidrug resistance. Characterization of the “resistance potential” of animal-derived and environmental samples is the first step toward incorporating metagenomic approaches into AMR surveillance in agricultural systems. Further research is needed to assess the public-health risk associated with different microbial resistomes.

## Introduction

Antimicrobial resistance (AMR) is an important worldwide public health issue (Penders et al., 2013; WHO, 2014). Due to public health concerns arising from potential of treatment failure in infected humans, there is a growing movement to produce food animals without antibiotics. In the dairy sector, the number of certified organic milk cows on U.S. farms increased from 38,000 in 2000 to 255,000 in 2011 (USDA-ERS, 2013). A recent consumer survey conducted by the Food Marketing Institute and the North American Meat Institute (FMI and NAMI, 2016) found that 40% of all respondents had purchased meat produced using organic or “natural” production practices within the last 3 months – an increase from 20% in 2007. Nevertheless, AMR is a ubiquitous feature in microbial populations and therefore, it is unsurprising that several scientific studies have detected AMR bacteria in organic and natural animal production systems where use of antibiotics was prohibited or restricted (Price et al., 2005; Cho et al., 2008; Kazimierczak et al., 2009).

Traditionally, AMR in bacterial communities has been investigated using cultures of indicator organisms such as *Escherichia coli*, *Campylobacter*, and *Salmonella* (Luangtongkum et al., 2006; Sapkota et al., 2014; Zwonitzer et al., 2016), or by amplification of a limited number of antibiotic resistance genes (ARGs; Cohen et al., 2012; Guarddon et al., 2014; Beukers et al., 2018) detected via polymerase chain reaction (PCR) methods. These approaches offer limited findings and conclusions because they select for only a few specific indicator bacterial species via enrichment and are limited to only a few ARGs. Furthermore, it is uncertain whether the presence of ARGs in specific taxa are representative of those within the entire microbial communities or the ARGs they contain (Gerzova et al., 2015). Recently, metagenomic approaches have been used to evaluate the impact of antimicrobial use on entire microbial communities (“microbiome” or “microbiota”) and their associated resistance determinants (“resistome”) in different agricultural environments (Agga et al., 2015; Noyes et al., 2016b; Yang et al., 2016). The main advantage of using metagenomics is the ability to look at the whole microbiome community and resistome in samples, improving our understanding of microbial communities (Noyes et al., 2016c). In the present study, we characterized the resistome and associated microbiome in feces and environmental samples of conventional and natural beef feedlot and dairy cattle production systems. We hypothesized that the resistome and microbial communities would differ between farms with different production practices and type of cattle. Increased understanding of the resistome in different livestock farms will aid our understanding of AMR ecology and discussing of effective production practices to limit ARGs dissemination from animals to humans.

## Materials and Methods

### Study Overview

An observational cross-sectional design was used for this investigation. Samples of cattle feces, wastewater from catchment basins, and soil where wastewater was applied were collected at a single time from a conventional feedlot, a natural feedlot, a conventional dairy, and an organic dairy. After DNA extraction and sequencing, shotgun metagenomic reads were aligned to a comprehensive reference database for identification of ARGs accessions (resistome) and bacterial taxa (microbiome) using Burrows–Wheeler Aligner (BWA) (Li and Durbin, 2009). Primary comparisons included differences in the resistomes associated with production practices (cattle raised conventionally vs. cattle raised without antibiotics; CONV vs. RWA), type of cattle (beef vs. dairy), and sample type (feces vs. soil vs. wastewater). To assess differences in resistome composition, comparisons were conducted using non-metric multidimensional scaling (NMDS) to ordinate samples and analysis of changes in abundance of individual classes, mechanisms, and groups of ARGs.

### Study Sites

A conventional feedlot (CONV-F), a conventional dairy (CONV-D), a natural feedlot (RWA-F), and an organic dairy (RWA-D) participated in the study. Feedlots were located in Alberta (Canada), separated by ∼500 km, with a capacity of 38,000 and 22,000 head in RWA-F and CONV-F, respectively. In the RWA-F, approximately half of the cattle were managed for “natural” beef production, while the other half was raised using conventional methods. Conventional and natural production systems were physically separated in the facility, including separate wastewater drainage and catchment basins. However, some conventional pens were in the natural area. Cattle raised in the natural feedlot had a branded program in compliance with the Canadian Food Inspection Agency guidelines for the label claims raised without added hormones, antibiotics, or animal by-products.

Dairy farms (CONV-D and RWA-D) were located in northern Colorado (United States), separated by ∼70 km, and both had a lactating herd of approximately 1,200 cows at the time of sampling. The RWA-D farm was certified as an organic producer by the United States Department of Agriculture National Organic Program requiring that animals were raised without the use of antibiotics, among other specifications. If antimicrobials had to be used to treat severe clinical illnesses or animal welfare issues in RWA animals, treated animals were removed from the natural (feedlot) or organic (dairy) program and placed in conventional pens.

### Sample Collection

Collection of soil and feces occurred in September 2015. The sampling plan, including the number of samples to take from each farm, was developed based on sequencing results from previous experiments in similar environments carried out by the same group of researchers (Noyes et al., 2016b, c; Yang et al., 2016; Zaheer et al., 2018). Fecal samples were collected from 16 pens in each feedlot, including 8 pens containing cattle that were on feed for a short period (13 ± 11 days) and 8 pens that were on feed for a long period (243 ± 38 days). Composite fecal samples (∼400 g/sample) were collected from the floor of each pen (one composite sample/pen) by pooling feces from 20 fresh fecal pats using sterile tongue depressors. Fecal samples were collected from two pens at both dairies; one with high producing lactating cows and the other with low producing cows. Eight composite fecal samples were collected from the floor of each dairy pen by pooling feces from 20 fresh fecal pats per composite sample. A total of 64 composite samples were collected, 32 from feedlots and 32 from dairies. After collection, fecal samples were immediately placed on ice and transported to a laboratory for stored at −80°C until DNA extraction. Antimicrobial treatments administered to conventionally housed feedlot (Supplementary Tables 1, 2) and dairy cattle (Supplementary Table 3) were recorded and reported.

Soil samples were collected from land where wastewater from the main catchment basins was used for irrigation of crops, including corn, sorghum, and canola. In the case of RWA-F, wastewater from the catchment basin receiving the effluents from the natural pens drained by overflowing into a larger basin containing effluents from both natural and conventional pens. Wastewater from this main catchment basin was applied to fields used to produce crops at the RWA-F. Wastewater was applied to the land by an irrigation pivot between 1 week (RWA-D) and ∼4–6 months (CONV-D, CONV-F, and RWA-F) before soil sampling based on basin capacity and crop requirements. In each field (∼2, 50, 65, and 150 ha for RWA-D, CONV-D, RWA-F, and CONV-F, respectively), eight composite soil samples (∼400 g/sample) were collected with a standard soil auger at a depth of 5–10 cm with each composite sample comprised of 20 individual soil cores. Fecal and soil samples were placed in Whirl-Pak bags (Nasco, Fort Atkinson, WI, United States) placed on ice and immediately transported to the laboratory where they were frozen at −80°C until DNA was extracted.

Catchment basins were sampled in September 2016, in the same season but 1 year after collecting fecal and soil samples. Although this temporal gap in the collection of samples may influence the results, the objective of this study was not following up the persistence and dissemination of ARGs within farms over time, but rather obtain a snapshot of the resistome in a given point in time. Eight water samples (500 ml each) were collected from each catch basin by walking along the edge of the catchment basins and taking sample at equal distances around the perimeter. Samples were collected 15–20 cm below the water surface using a sampling pole (Nasco, Fort Atkinson, WI, United States) and 500 ml sterile plastic containers (Thomas Scientific, Swedesboro, NJ, United States). Bottles were transported to the laboratory, and on the same day of collection, the entire 500 ml volume from each sample was centrifuged at 10,000 ×*g* for 20 min at 4°C. The resultant pellet was stored at −80°C until DNA was extracted.

### DNA Extraction and Metagenomic Sequencing

Ten grams of thawed feces from each sample were weighed, suspended in buffer peptone water, and allowed to sediment to separate bacterial cells from heavy particulates and debris as described by Noyes et al. (2016c). For soil samples, 10 g of thawed soil was weighed from each sample with no sedimentation. The DNA was extracted directly from soil samples and from fecal and water pellets (1.5–8.0 g) using the PowerSoil^®^ DNA isolation kit (Mo Bio Laboratories, Inc., CA, United States) according to the manufacturer’s protocol.

After DNA extraction and ethanol precipitation, DNA concentration and quality were measured using the NanoDrop 1000 Spectrophotometer (Thermo Fisher Scientific, Germany) and a Qubit^TM^ assay (Thermo Fisher Scientific, Germany). Two micrograms of DNA (40–50 μl) from each sample were transported on ice to the Genomics and Microarray Core at the University of Colorado–Denver (Denver, CO, United States). Genomic libraries were prepared using the Illumina TruSeq DNA PCR-Free LT Library Kit (Illumina, Inc., San Diego, CA, United States). Paired-end sequencing (2 × 150 bp) was performed on the Illumina HiSeq 4000 HT sequencing platform with 6 soil, 8 feces, and 16 wastewater samples per lane. The required sequencing depth needed to characterize resistomes in different type of samples was hard to estimate as this study was one of the first aiming to a metagenomic comparison of the resistome between different habitats. As a reference, we took results reported by Zaheer et al. (2018) who demonstrated that ∼50 million reads would be a suitable compromise for sequencing bovine fecal samples and adequately inferring their resistome. Then, we expected a greater and lower resistome diversity in soil and wastewater, respectively, compared to feces, adjusting the desired number of reads per sample according to that (>50 million reads for soil samples and <50 million reads for wastewater samples).

Raw sequencing data for all 128 samples in the present study are publicly available at the National Center for Biotechnology Information (NCBI) database with study accession number SRP 109087.

### Data Processing

Shotgun sequencing data were analyzed using AmrPlusPlus (Lakin et al., 2016). In brief, raw sequencing reads were trimmed using Trimmomatic version 0.36 (Bolger et al., 2014) and bovine DNA sequences were removed by aligning the trimmed sequences to reference cattle genomes (Zimin et al., 2009; Canavez et al., 2012) using BWA version 0.6.2 (Li and Durbin, 2009). For detection of ARGs, remaining sequences were aligned using BWA to approximately 4000 hand-curated ARGs contained in the MEGARes database version 1.01 (Lakin et al., 2016). Individual ARGs were defined as published sequences with unique accession numbers in public databases. The MEGARes database includes structural genes with regulatory activities as the primary function (i.e., efflux pumps), although they are usually chromosomally encoded and do not necessarily determine functional resistance phenotypes. However, several reviews have reported a link those regulatory genes and AMR, especially in pathogens (Piddock, 2006; Olivares et al., 2013; Blanco et al., 2016). ARGs were aggregated and classified hierarchically to three levels: class (e.g., tetracycline), mechanism (e.g., ribosomal protection proteins), and group (e.g., TetQ) (Lakin et al., 2016). The group annotation provides information on the major gene category preserving the nucleotide identity within groupings and maintaining reasonable biological categories across the database. Only individual ARGs that were covered >80% in length by sample reads, and those where resistance was not conferred by single nucleotide polymorphism were considered for downstream analysis. Phylogenetic classifications were assigned to trimmed microbial sequences using Kraken (version 0.10.6-beta), which uses the NCBI reference nucleotide database (RefSeq) to classify bacteria at different taxonomic levels (Wood and Salzberg, 2014). In this study, results are presented at the phylum level. Antibiotic resistance and microbial count tables were normalized using a cumulative sum scaling (CSS) method (Paulson et al., 2013). Compared to total sum scaling (TSS, the most common normalization approach), where read counts from each gene are divided by the total number of read counts in each individual sample, CSS basically do the same but, in the denominator uses the total number of read counts starting from low-abundant genes up to a threshold in order to minimize the influence of variable high-abundant genes. Buongermino Pereira et al. (2018) reported that CSS had among the best performance for large metagenomic datasets comparing nine normalization methods for count data.

### Statistical Analysis of Sequencing Data

A zero-inflated Gaussian distribution mixed model native to the metagenomeSeq R package (Paulson et al., 2013) was used to evaluate differential abundance in features of the microbiome and resistome. *A priori* primary comparisons included resistome and microbiome differences between CONV and RWA samples; between feedlot and dairy farms; and differences among type of samples (feces, wastewater, and soil). Statistical inferences for each feature occurred after log_2_ transformation, followed by a multiple-comparison Benjamini–Hochberg adjustment using a critical α = 0.05. Data were visualized in NMDS ordination plots and statistical inference (α = 0.05) was made using the analysis of similarity (ANOSIM) included in the vegan package (version 2.2-2; Oksanen et al., 2014). The ANOSIM *R*-value ranged from 0 to 1 with 0 indicating total similarity and 1 total dissimilarity. Procrustes (Peres-Neto and Jackson, 2001) and Protest (Jackson, 1995) included in vegan R package were used to compare congruence of the microbiome and resistome ordinations based on α = 0.05, correlation coefficient (*r*), and measure of fit (*m*^2^). Richness (i.e., number of unique mechanisms of resistance counted in a sample) and Shannon’s diversity index (i.e., number and proportion of unique mechanisms of resistance counted in a sample) were calculated using vegan package version 2.2-2 (Oksanen et al., 2014).

## Results

### Sequencing Results

Shotgun metagenomic sequencing yielded 5.45 billion reads; 52, 35, and 13% of those sequences were obtained from feces, soil, and wastewater samples, respectively. Metagenomic sequencing produced (mean ± SD) ∼45 ± 16 million raw reads per fecal sample, and 60 ± 25 and 22 ± 9 million reads per soil and wastewater samples, respectively. Given these difference in sequencing depth (reads per sample), comparisons between type of samples (feces vs. soil vs. wastewater) should be carefully interpreted, even after normalization, as deeper sequencing results in a greater probability of finding ARGs. The mean quality Phred score of reads was 35.3 in feces (*n* = 64; min. 33.8; max. 37.2), 35.2 in soil (*n* = 32; min. 31.2; max. 37.8), and 31.3 in wastewater samples (*n* = 32; min. 28.9; max. 33.2). Only 3.96 million raw reads, out of the total sequenced reads (5.45 billion reads), were successfully aligned to the MEGARes database and used for the downstream analysis of resistomes. After normalization, the number of reads (mean ± SD) per sample aligned to ARGs was 877 ± 297, 501 ± 391, and 285 ± 109, for feces, wastewater, and soil samples, respectively. A total of 440 individual ARGs were identified and assigned to 16 classes, 44 mechanisms, and 192 groups of AMR determinants (Supplementary Table 4). In addition, 5.7% of the reads were aligned to microbial sequences present in the Kraken database. Supplementary Table 5 provides a summary of the total number of reads sequenced and the proportion of these aligned to databases which were used for downstream analysis.

### Differences in the Resistome Between Conventional vs. Raised Without Antibiotic

The average number of individual ARGs identified per sample was greater (*P* < 0.05) in samples obtained from CONV production systems (ARGs = 77, min. 2, max. 260) than in samples obtained from RWA farms (ARGs = 51, min. 0, max. 126). This was a consequence of differences among feces (118 and 79 ARGs per sample in CONV and RWA, respectively; *P* < 0.05) and wastewater (47 and 22 ARGs per sample in CONV and RWA, respectively; *P* < 0.05) samples.

**FIGURE 1:**
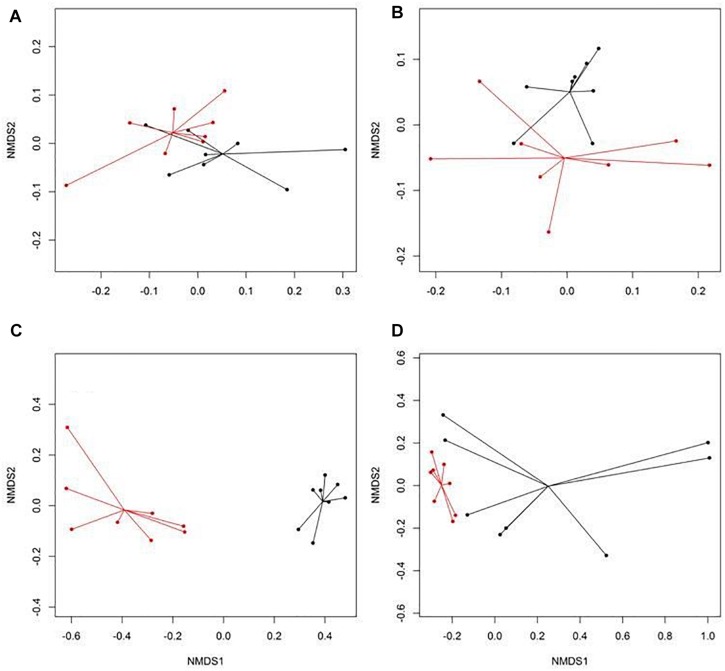
Non-metric multidimensional scaling (NMDS) ordination of the resistomes at the mechanism level for **(A)** feces collected from feedlot cattle early in the feeding period, **(B)** feces collected from feedlot cattle late in the feeding period, **(C)** wastewater (WW), and **(D)** soil samples separated by production practices (black: conventional, CONV; red: natural, NAT) in feedlots.

**FIGURE 2:**
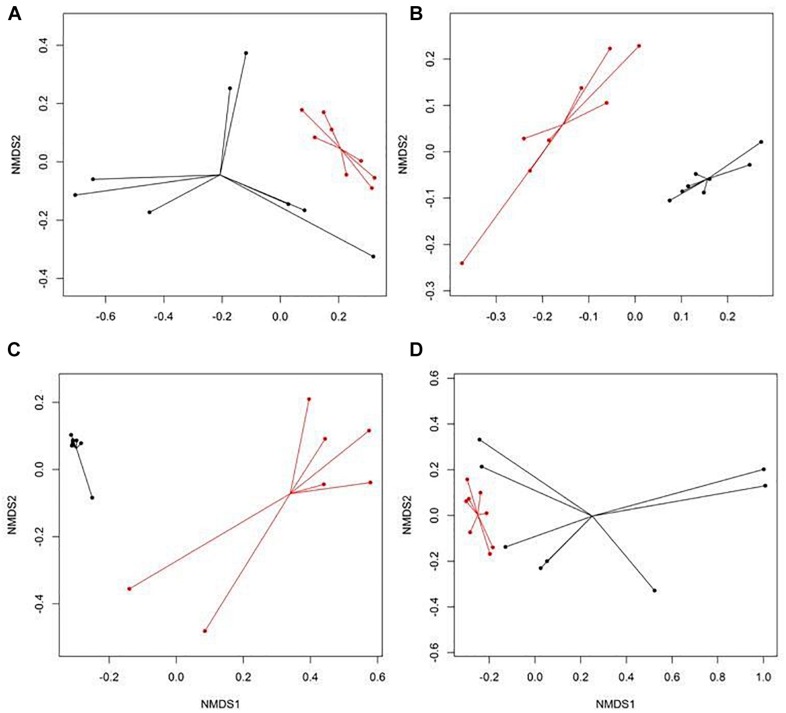
Non-metric multidimensional scaling (NMDS) ordination of the resistomes at the mechanism level for **(A)** feces collected from low producing dairy cows, **(B)** feces collected from high producing dairy cows, **(C)** wastewater (WW), and **(D)** soil samples separated by production practices (black: conventional, CONV; red: organic, ORG) in dairy farms.

There were eight NMDS pairwise comparisons between CONV and RWA samples (two types of cattle × four types of samples). The NMDS ordination plots demonstrated significant (ANOSIM *P* < 0.05, stress < 0.06) separation of CONV vs. RWA resistomes for feces collected from late feeding pens and wastewater in feedlots (Figure 1) and for wastewater, soil, and feces collected from low and high producing dairy cows in dairies (Figure 2). Ordination plots were presented at the mechanism level of resistance, with similar results at class and group level (Supplementary Table 6). Wastewater samples showed the greatest resistome separation (*P* < 0.05, ANOSIM *R* > 0.80) between CONV and RWA samples (Figures 1C, 2C) at all levels of resistance. Among feces, separation of CONV and RWA resistomes was greater for feces collected from late feeding pens in feedlots (Figure 1B; ANOSIM *R* = 0.37) and from high producing cows in dairies (Figure 2B; ANOSIM *R* = 0.81). In soil, differences in resistome composition (ANOSIM *P* < 0.05) were detected between CONV and RWA samples in dairy farms, but with low extent of separation (ANOSIM *R* = 0.24).

**FIGURE 3:**
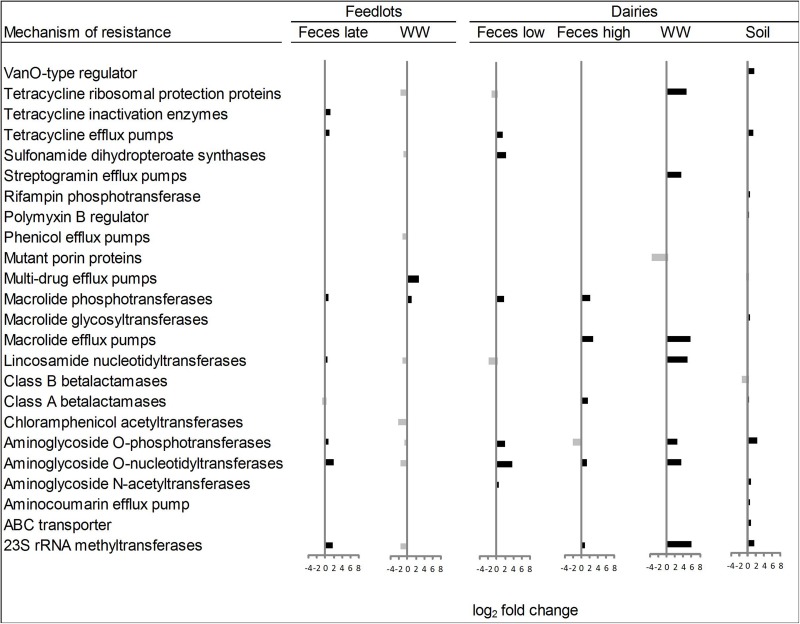
Mechanisms of resistance more abundant (*P* < 0.05) in conventional (right bars, black) or in “raised without antibiotic” farms (left bars, gray), for types of sample that showed significant (*P* < 0.05) non-metric multidimensional scaling (NMDS) separation in ordination plots (Feces late, feces collected from feedlot cattle late in feeding period; Feces low, feces collected from low producing dairy cows; Feces high, feces collected from high producing dairy cows, and WW, wastewater).

Within the six ordination plots that were different (ANOSIM *P* < 0.05) among CONV and RWA resistomes, 24 mechanisms of resistance, out of 44 identified, were associated with production practices (Figure 3). Twelve mechanisms were more abundant (*P* < 0.05) in CONV samples (e.g., tetracycline efflux pumps and macrolide phosphotransferases), four in RWA samples (e.g., class B lactamases and mutant porin proteins), and the abundance of eight mechanisms varied depending on sample type (e.g., tetracycline ribosomal protection proteins and 23S rRNA methyltransferases). At the group level, out of 192 resistance genes, 29 that were uniquely identified in samples collected from CONV feedlots or dairies (e.g., not on RWA farms), but these were of relatively low abundance accounting for ∼1% of the total reads aligned to ARGs (Supplementary Table 7). Those unique groups were found in few CONV samples (<9 out of 64 total samples) except for OXA (D-β-lactamase), ERM (23S rRNA methyltransferase), and LNUF (lincosamide nucleotidyltransferase), which were identified in 23, 17, and 16 CONV samples, respectively.

### Differences in Resistome Composition Associated With Feces vs. Environment Samples

Fecal samples contained more (*P* < 0.05) ARGs (average 99 per sample, min. 20, max. 260) than soil (average 24 per sample, min. 2, max. 89) and wastewater samples (average 35 per sample, min. 0, max. 92). Resistance to sulfonamide was identified in feces (12% of samples) and wastewater (53% of samples) but was not detected in soil. On the other hand, ARGs conferring resistance to rifampin and aminocoumarin were detected in 94 and 81% of soil samples, respectively, but were not in feces or wastewater. Wastewater shared 62 resistance groups (83%) with feces, but only 21 with soil samples (28%). Only 16 resistance groups (8%) were shared among feces, wastewater, and soil corresponding to multidrug (MexK, HNS, Msr, Sme, EmrE), tetracycline (TetA, TetL, TetX, TetZ), MLS (MphE, MyrA, Erm), aminoglycoside (Aph6, Aph3-dprime), phenicol (Cat), and β-lactam (AmpR) classes of resistance.

**FIGURE 4:**
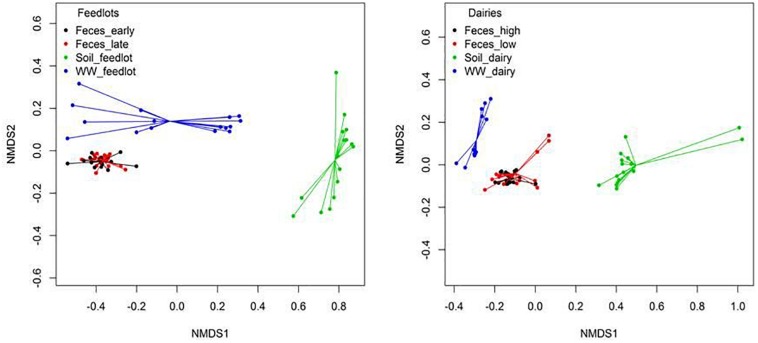
Non-metric multidimensional scaling (NMDS) ordination plot showing resistome differences at the mechanism level between feces, soil, and wastewater (WW) samples in feedlot (ANOSIM *P* < 0.05, ANOSIM *R* = 0.67, stress = 0.03) and dairies (ANOSIM *P* < 0.05, ANOSIM *R* = 0.74, stress = 0.05). Similar results were obtained at class (Feedlot: ANOSIM *P* < 0.05, ANOSIM *R* = 0.65, stress = 0.02, Dairy: ANOSIM *P* < 0.05, ANOSIM *R* = 0.67, stress = 0.05) and resistance groups (Feedlot: ANOSIM *P* < 0.05, ANOSIM *R* = 0.72, stress = 0.01, Dairy: ANOSIM *P* < 0.05, ANOSIM *R* = 0.80, stress = 0.06).

Clustering of resistomes in ordination plots was influenced by sample type in both feedlots and dairies (Figure 4). Resistance to tetracyclines and MLS was more abundant (*P* < 0.05) in feces than in environmental samples. In soil samples, classes conferring resistance to multiple drugs, rifampin, aminocoumarins, and glycopeptides were more abundant (*P* < 0.05) than in feces and wastewater samples. Hits to tetracycline, MLS, and sulfonamide class drugs were the most prevalent sequences identified in wastewater samples. Feces, wastewater, and soil samples had hits to 49 (41%), 8 (11%), and 60 (67%) unique groups of resistance, respectively (e.g., groups detected only in feces but not detected in wastewater or soil). Supplementary Table 8 shows the 10 most abundant unique groups for each sample type.

### Differences in Resistome Composition Associated With Type of Cattle; Feedlot vs. Dairy Farms

Across all sample types, the average number of ARGs identified per sample was greater (*P* < 0.05) in feedlot (76 ARGs; min. 6, max. 260) than in dairy samples (52 ARGs; min. 0, max. 151). This tendency was observed for feces (112 and 86 ARGs, feedlots and dairies, respectively; *P* < 0.05), wastewater (53 and 117 ARGs; *P* < 0.05), and soil (29 and 20 ARGs, *P* > 0.05). In comparisons between CONV farms (CONV-F vs. CONV-D) and RWA farms (RWA-F vs. RWA-D), tetracycline and MLS classes of resistance were more abundant (*P* < 0.05) in feedlot cattle than in dairy cow feces, whereas the β-lactam class of resistance was more abundant (*P* < 0.05) in dairy cow feces. When comparing feedlot and dairy wastewater samples resistance to tetracyclines and multi-drugs was more abundant (*P* < 0.05) in wastewater collected from feedlots; while resistance to MLS was more prevalent (*P* < 0.05) in dairy wastewater samples. Fewer differences were observed in the resistome of soil samples between feedlot and dairy farms; only glycopeptide and cationic antimicrobial peptides resistance were more abundant (*P* < 0.05) in RWA-F soil than in RWA-D.

### Microbial Communities Associated With the Different Resistomes

In general, weak correlations and high dissimilarities were found between resistome and microbiome ordinations (Supplementary Table 9). Based on procrustes analysis, the strongest relationship between ARGs and the microbial community was found in samples collected from cattle late in the feeding period (*n* = 16; *P* = 0.06, *r* = 0.59, *m*^2^ = 0.65). Microbial communities were dominated by bacteria belonging to *Proteobacteria*, *Actinobacteria*, *Firmicutes*, and *Bacteroidetes*, which accounted for 96.6% of the total aligned normalized reads at phylum level (Supplementary Table 10). Similar to the resistome, there were eight pairwise comparisons between CONV and RWA microbiomes, one for each combination of farm and sample type. The NMDS ordination plots demonstrated separation (ANOSIM *P* < 0.05) of CONV and RWA microbiomes in feces collected from beef cattle early vs. late in the feeding period (ANOSIM *R* = 0.42 and 0.24, respectively), and feedlot wastewater (ANOSIM *R* = 0.87). Among dairies, microbiome differences (*P* < 0.05) were identified between CONV and RWA in feces collected from low producing cows, soil, and wastewater (ANOSIM *R*: 0.70, 0.36, and 0.98, respectively).

Feces, wastewater, and soil samples clustered apart in the NMDS ordination plot in feedlots (ANOSIM *P* < 0.05, *R* = 0.83, stress: 0.03) and dairies (ANOSIM *P* < 0.05, *R* = 0.64, stress: 0.06). *Proteobacteria* and *Actinobacteria* increased (*P* < 0.05) in the soil microbiome relative to the fecal and wastewater microbiome. Conversely, *Bacteroidetes* and *Firmicutes* were more abundant (*P* < 0.05) in feces compared to soil and wastewater. Wastewater samples had a greater abundance (*P* < 0.05) of *Proteobacteria* than feces.

### Resistome and Microbiome Ecological Indexes

Samples collected from CONV vs. RWA, feces vs. soil and wastewater, and samples collected from feedlots vs. dairy farms tended to show greater richness (number of different resistance determinants per sample) at class, mechanism, and group resistance (Supplementary Table 11). With regard to Shannon’s diversity index, which accounts for both abundance and evenness of resistance determinants per sample, feces from dairy cows had the greatest resistome diversity at class, mechanism, and group level. In the microbiome, only the type of sample had an effect (*P* < 0.05) on the Shannon’s diversity index (feces: 1.4–1.6; wastewater: 1.0; soil: 0.9).

## Discussion

This study demonstrated that resistomes in beef feedlot and dairy cattle operations were different between farms with distinct production practices (CONV vs. RWA). Although this agrees with results reported by Vikram et al. (2017) who found slight differences in the fecal resistome of CONV and RWA cattle, it cannot be discounted that between-farm variability inherent to resistomes may have influenced the differences measured in this study. For that reason, results can only relate to the individual farms sampled in the present study and extrapolation to other farms would be speculative. Although the limitations, data from this work demonstrate the ability of metagenomics to detect multiple ARGs in microbial communities in the context of commercial farms. As the cost of next generation sequencing decreases, future studies should sample more farms thus reduce the likelihood that individual location would influence comparisons. Environment, diet, cattle source, management practices, and location of the farms have been previously cited as confounding factors in attempt to ascertain the relationship between antibiotic use and AMR prevalence (Singer et al., 2006; Singer and Williams-Nguyen, 2014; Benedict et al., 2015) and, more importantly, its dissemination and risk to human health. In a similar study with swine, geographical location in which the swine were raised had a greater impact in defining microbial resistomes than differential use of antibiotics in conventional and organic farms (Gerzova et al., 2015). Recently, Doster et al. (2018) reported that transition of calves into the feedlot – and associated changes in diet, geography, conspecific exposure, and environment – may exert a greater influence over the fecal resistome and microbiome of feedlot cattle than common antimicrobial drug treatments. In contrast, a clinical trial on feedlot cattle found that prolonged antimicrobial exposure was linked to an increase of that class of resistance (Weinroth et al., 2018). In our study, lack of congruence between ARGs and microbial communities in CONV and RWA farms (procrustes analysis) suggested that other factors, in addition to antimicrobial use, were associated with changes in resistome and microbiome composition. Also, it may reflect the possibility that a small fraction of the total bacterial population was resistant to antibiotics (McLean and Vogwill, 2015; Xiao et al., 2016), and that horizontal gene transfer disrupted the link between microbiome and resistome composition (Forsberg et al., 2014; Johnson et al., 2015).

Overall, only ∼4 million reads (∼0.7%), of the total 5.45 billion reads across all dataset, were associated with AMR. Hits to different mechanisms of resistance were generally more abundant (total abundance) in feces from conventionally raised cattle, suggesting that exposure to antibiotics can promote the mobilization and dissemination of AMR among microbial communities through horizontal gene transfer and/or clonal expansion of resistant taxa (Looft et al., 2012; Perry and Wright, 2013; Chambers et al., 2015). However, feces of animals raised without antibiotics harbored a diverse resistome. This finding was consistent with data from other studies, where ARGs were identified in the feces of cattle that were not exposed to antibiotics (Reinstein et al., 2009; Morley et al., 2011; Santamaria et al., 2011). It supports the concept that AMR is an ancient phenomenon that does not depend, alone, on antimicrobial use to emerge (D’Costa et al., 2011; Stokes and Gillings, 2011). Some classes of resistance (e.g., aminoglycosides) were found in conventional samples, even though farm managers did not report using those antimicrobial drugs. This emphasized the idea that ARGs can be selected and enriched without using the respective antimicrobial, either through co-selection of resistance genes or through selection of some portions of the microbiome which contain these resistance genes. Similarly, the fact that some resistance mechanisms were more abundant in RWA samples than in CONV samples could be related to the natural presence of ARGs within the microbiomes associated with these environments (Noyes et al., 2016a), but further investigation is required. Independent of antibiotics used, diverse ARGs may be ubiquitously distributed in nature and maintained because of their co-localization in complex resistance clusters, including genes coding for antibiotic, metal, and biocide resistance (Stokes and Gillings, 2011; Johnson et al., 2015; Pal et al., 2015). It is known that higher concentrations of heavy metals (zinc, copper) usually included in the diet of RWA cattle compared to CONV cattle to replace antibiotics may result in the emergence of bacterial populations co-resistant to metal and antibiotics (Reinstein et al., 2009; Pal et al., 2015). For these reasons, it has been hypothesized that ARGs may persist in various types of environments despite discontinued use of antimicrobials (Johnson et al., 2015).

Feces from beef cattle had a more abundant resistome than those from dairy cattle, as measured by the number of AMR reads and unique ARGs identified in each sample. Antimicrobial drugs are not included in diets of dairy cows as milk produced from antibiotic-treated cows must be discarded, depending on withholding time for each drug, to prevent antibiotic residues from entering the food chain (Government Accountability Office, 2011). Abundance of ARGs in feces from feedlots was greater for antibiotics that are commonly used as in-feed ingredients, such as tetracyclines and macrolides, while the resistome of feces from dairies contained mainly sequences aligning to β-lactams. Tetracyclines have been used in agricultural environments for decades in North America. This suggests a theoretical link between tetracycline use and enrichment of bacterial genes conferring resistance to tetracyclines, and that stop using tetracyclines in RWA farms would not cause an automatic reduction in resistance. The RWA farms visited in the present study stopped using antibiotics in animals 10 and 14 years ago (feedlot and dairy, respectively), but even so, tetracyclines resistances genes still dominated the fecal resistome of these samples. On the other hand, resistance genes of major public health concern were detected at lower abundance in the present study, such as genes encoding β-lactamases (i.e., class A in feces and class B in wastewater) and fluoroquinolone resistance efflux transporter proteins in soil. Because cephalosporins and fluoroquinolones have been approved with restrictions for use in cattle, these genes could be readily enriched in the presence of direct selection pressure (as it happened with tetracyclines) recommending a prudent use of antibiotics to prevent the rise and transfer of critical ARGs from livestock farms.

Our results suggested that, in feces, the resistome was associated with exposure to antibiotics commonly used in veterinary medicine, whereas the soil resistome was influenced more by diverse and naturally occurring mechanisms of resistances (Sengupta et al., 2013). Coincidently, the soil microbiome was dominated by *Proteobacteria* and *Actinobacteria*, which are known for carrying ARGs classified as drug transporters (e.g., efflux pumps) to cope with the variable exposures found in natural environments (Forsberg et al., 2014) when compared to fecal samples which were dominated by *Bacteroidetes* and *Firmicutes*. Feces, wastewater, and soil microbiomes clustered apart in the microbiome ordination plot, meaning that the composition of the microbiome differed among these environments and could present a taxonomic barrier for exchange of ARGs (Hu et al., 2016). There is concern that land application of wastewater could enrich for antimicrobial resistant bacteria in soils due to the presence of antibiotic residues in cattle manure (Cytryn, 2013; Kyselkova et al., 2015; Pornsukarom and Thakur, 2016). Recently, Udikovic-Kolic et al. (2014) and Hu et al. (2015) found that application of manure from cattle that were not exposed to antibiotics increased antibiotic-resistant bacteria in soils, suggesting that manure-derived nutrients exert a strong selection pressure on soil bacteria and ARG composition, irrespective of antibiotics used in animals. Presence of ARGs in catch basins may be influenced by antibiotics used in animals, but also by environmental conditions that affect the fate of bacteria harboring AGRs after release into the environment (Peak et al., 2007). Previous research suggests that wastewater from CONV-F and swine farms are more likely to contain higher abundance of ARGs compared to organic farms (Jindal et al., 2006; Peak et al., 2007). However, in our study, we could not determine whether differences in manure and wastewater resistomes were due to antibiotic use or to confounding factors (geographic location or effluent management systems) that were not specifically controlled by the study design. But, as it was stated before, our objective was to obtain a snapshot of microbial resistomes of different commercial farms.

These data demonstrated that shotgun metagenomics has a promising future for AMR surveillance because it takes into account the entire microbial community, however, underreporting may occur due to limitations in sequencing depth, especially in low abundance genes (Burcham et al., 2019). This is aggravated by the low proportion of metagenomic reads aligned to databases found in this work and in other studies that reported similar levels (Fitzpatrick and Walsh, 2016; Noyes et al., 2016c). For abundant ARGs (i.e., conferring resistance to tetracyclines), Vikram et al. (2017) reported a strong correlation between metagenomics and qPCR in the proportion of positive samples; however, qPCR was more sensitive for low-abundance genes (i.e., conferring resistance to β-lactams). Similarly, Weinroth et al. (2018) reported that if rare ARGs are more important to surveillance than common ARGs, then PCR is a better tool for interrogating AMR ecology than shotgun sequencing. Other limitations of the metagenomic approach are that mapping short reads to databases does not provide information about the bacterial host (commensal or pathogenic) and the localization of the resistance gene in the bacterial genome (chromosome or mobile genetic element). This kind of contextual information of ARGs provides a better characterization of microbial resistomes and their associated human health risk (Port et al., 2014; Fitzpatrick and Walsh, 2016; Crofts et al., 2017). Furthermore, presence of ARGs does not mean they are expressed and able to confer AMR. Forslund et al. (2013) proposed the term “resistance potential” instead of “resistance” to reflect differences in gene expression and regulation that can affect phenotypic resistance. Characterization of the “resistance potential” of animal-derived and environmental samples is the first step toward incorporating metagenomic approaches into AMR surveillance in agricultural systems.

## Data Availability

The datasets generated for this study can be found in NCBI accession SRP109087 https://www.ncbi.nlm.nih.gov/sra/SRP109087.

## Author Contributions

KB and PM designed this study, obtained funding, secured partnership with commercial farms where the study wasconducted, and provided oversights to all other aspects of the study. PM, KB, TM, CWB, and PR collaborated on study design and sampling strategies. PR and XY collected the samples. PR, SC, ED, MW, and JP performed the sample processing. PR, SL, ED, NN, and CB oversaw and performed the bioinformatic analysis. All authors read, edited, and approved the final manuscript.

## Conflict of Interest Statement

CWB is part owner and managing partner of Feedlot Health Management Services and Southern Alberta Veterinary Services. Feedlot Health is a private company that provides expert consultation regarding production and management of feedlot cattle and calf grower calves, including developing veterinary protocols to support animal health. They also conduct in-house and contract research related to dairy calf grower and feedlot production. The remaining authors declare that the research was conducted in the absence of any commercial or financial relationships that could be construed as a potential conflict of interest.
