# Supplementary material for: Characterization of the Microbial Resistome in Conventional and “Raised Without Antibiotics” Beef and Dairy Production Systems
Source: Front Microbiol. 2019 Sep 4;10:1980. doi: 10.3389/fmicb.2019.01980 (PMC6736999; doi:10.3389/fmicb.2019.01980)
Supplement: Supplementary file 1 [file Table_1.DOCX]

Supplementary Table 1. Antimicrobial usage in early feeding pens (n = 8) in the conventional feedlot.

|  |  | **Days on feeding (d) and animals/pen (a) at the time of sampling** | | | | | | | | | | | |
| --- | --- | --- | --- | --- | --- | --- | --- | --- | --- | --- | --- | --- | --- |
|  |  | 1 d 274 a | 5 d 262 a | 5 d 268 a | | 8 d 264 a | | 8 d 276 a | | 22 d 282 a | 29 d 270 a | 29 d 284 a |  |
| **Antimicrobial ingredient (Dose equivalent)** |  | **Percentage of animals treated since the beginning of feeding** | | | | | | | | | | |  |
| Monensin sodium (25 mg/kg diet DM^1^) |  | 100 | 100 | 100 | 100 | | 100 | | 100 | | 100 | 100 |  |
| Chlortetracycline (35 mg/kg diet DM) |  | 100 | 100 | 100 | 100 | | 100 | | 100 | | 100 | 100 |  |
| Ceftiofur sodium (1.0 mg/kg BW^2^) |  | 0 | 0.4 | 0 | 5.3 | | 0 | | 0.35 | | 1.1 | 3.5 |  |
| Florfenicol (40 mg/kg BW) |  | 6.2 | 0 | 0 | 2.3 | | 6.5 | | 0 | | 0 | 1.8 |  |
| Oxytetracyline (20 mg/kg BW) |  | 97.8 | 100 | 100 | 100 | | 98.5 | | 100 | | 100 | 100 |  |
| Tulathromycin (2.5 mg/kg BW) |  | 0 | 0 | 0 | 0 | | 0 | | 0 | | 0 | 0.7 |  |
| Trimethoprin (2.67 mg/kg BW), Sulfadoxine (13.33 mg/kg BW) |  | 0 | 0 | 1.1 | 4.5 | | 2.2 | | 1.1 | | 0 | 2.1 |  |
| Sulfanilamide (73.13 mg/kg BW), Sulfathiazole (73.13 mg/kg BW), Sulfamethazine (48.75 mg/kg BW) |  | 0 | 0 | 0 | 0.38 | | 0 | | 0 | | 0 | 0 |  |

^1^DM: Dry Matter

^2^BW: Body Weight
