# Supplementary material for: Characterization of the Microbial Resistome in Conventional and “Raised Without Antibiotics” Beef and Dairy Production Systems
Source: Front Microbiol. 2019 Sep 4;10:1980. doi: 10.3389/fmicb.2019.01980 (PMC6736999; doi:10.3389/fmicb.2019.01980)
Supplement: Supplementary file 2 [file Table_2.DOCX]

Supplementary Table 2. Antimicrobial usage in late feeding pens (n = 8) in the conventional feedlot.

|  |  | | **Days on feeding (d) and animals/pen (a) at the time of sampling** | | | | | | | | | | | | |
| --- | --- | --- | --- | --- | --- | --- | --- | --- | --- | --- | --- | --- | --- | --- | --- |
|  |  | | 181 d 93 a | | 215 d 206 a | 233 d 272 a | | 240 d 284 a | | 244 d 45 a | | 246 d 270 a | 286 d 83 a | 301 d 261 a |  |
| **Antimicrobial ingredient and dose equivalent** | |  | | **Percentage of animals treated since the beginning of feeding** | | | | | | | | | | |  |
| Monensin sodium, 25mg/kg DM^1^ | | |  | 100 | 100 | 100 | 100 | | 100 | | 100 | | 100 | 100 |  |
| Chlortetracycline, 35 mg/kg DM | | |  | 100 | 100 | 100 | 100 | | 100 | | 100 | | 100 | 100 |  |
| Chlortetracycline, 1 g/head/d | | |  | 100 | 0 | 0 | 100 | | 100 | | 100 | | 100 | 100 |  |
| Chlortetracycline, 6 g/head/d | | |  | 100 | 0 | 0 | 100 | | 100 | | 100 | | 100 | 100 |  |
| Ceftiofur sodium,1.0 mg/kg BW^2^ | | |  | 8.6 | 21.8 | 25.4 | 23.6 | | 6.7 | | 20.4 | | 10.8 | 23.0 |  |
| Ceftiofur crystalline free, 6.6 mg/kg BW | | |  | 0 | 0 | 1.5 | 1.8 | | 4.4 | | 3.3 | | 9.6 | 7.7 |  |
| Enrofloxacin, 7.7 mg/kg BW | | |  | 0 | 0 | 0 | 0 | | 0 | | 0 | | 3.6 | 0.38 |  |
| Florfenicol, 40 mg/kg BW | | |  | 9.7 | 0 | 0 | 0.35 | | 0 | | 1.1 | | 6.0 | 5.4 |  |
| Oxytetracyline, 20 mg/kg BW | | |  | 8.6 | 2.4 | 9.2 | 3.2 | | 48.9 | | 35.6 | | 57.8 | 10.3 |  |
| Oxytetracyline, 30 mg/kg BW | | |  | 92.4 | 0 | 0 | 0 | | 0 | | 0 | | 1.2 | 0 |  |
| Oxytetracyline ,6.67 mg/kg BW | | |  | 9.7 | 0 | 0 | 0 | | 0 | | 0 | | 0 | 0 |  |
| Tylosin tartrate, 29 mg | | |  | 100 | 100 | 100 | 100 | | 100 | | 100 | | 100 | 100 |  |
| Tulathromycin, 2.5 mg/kg BW | | |  | 1.1 | 100 | 0.37 | 100 | | 100 | | 100 | | 100 | 100 |  |
| Trimethoprin (2.67 mg/kg BW) and Sulfadoxine (13.33 mg/kg BW) | | |  | 6.5 | 2.9 | 8.8 | 12.7 | | 0 | | 5.5 | | 7.2 | 10.3 |  |

^1^DM: Dry Matter

^2^BW: Body Weight.
