# Supplementary material for: Characterization of the Microbial Resistome in Conventional and “Raised Without Antibiotics” Beef and Dairy Production Systems
Source: Front Microbiol. 2019 Sep 4;10:1980. doi: 10.3389/fmicb.2019.01980 (PMC6736999; doi:10.3389/fmicb.2019.01980)
Supplement: Supplementary file 3 [file Table_3.DOCX]

Supplementary Table 3. Antimicrobial usage in low and high producing pens in the conventional dairy farm (total number of cows per pen at sampling time: ̴ 130 cows).

|  |  |  | **Number of cows treated**^1^ | |
| --- | --- | --- | --- | --- |
| **Antimicrobial** | **Class** | **Dose equivalent** | **Low producing pen** | **High producing pen** |
| Ceftiofur crystalline free acid | Beta-lactam | 6.6 mg/kg BW^2^ | 16 | 23 |
| Ceftiofur hydrochloride | Beta-lactam | 2.2 mg/kg BW | 21 | 16 |
| Ceftiofur hydrochloride | Beta-lactam | 125 mg per quarter | 15 | 21 |
| Ampicillin | Beta-lactam | 6.0 mg/kg BW | 90 | 32 |

^1^ Number of cows present in the pen at the time of sampling and treated with corresponded drug at least one year before our sampling date (08/31/2014 – 09/01/2015).

^2^BW: Body Weight
