# Supplementary material for: Characterization of the Microbial Resistome in Conventional and “Raised Without Antibiotics” Beef and Dairy Production Systems
Source: Front Microbiol. 2019 Sep 4;10:1980. doi: 10.3389/fmicb.2019.01980 (PMC6736999; doi:10.3389/fmicb.2019.01980)
Supplement: Supplementary file 6 [file Table_6.DOCX]

Supplementary Table 6. Analysis of similarities (ANOSIM) statistics on the comparison of resistome compositions in samples collected from farms adopting conventional versus raised without antibiotics^1^.

|  |  | Antimicrobial resistance level | | | | | | |
| --- | --- | --- | --- | --- | --- | --- | --- | --- |
|  |  | Class | | |  | Group | | |
|  |  | *P*^2^ | R^3^ | Stress^4^ |  | *P* | R | Stress |
| **Feedlots** |  |  |  |  |  |  |  |  |
| Feces from early on feeding pens |  | 0.69 | 0.00 | 0.04 |  | 0.00 | 0.24 | 0.09 |
| Feces from late on feeding pens |  | **0.03** | 0.18 | 0.02 |  | **0.00** | **0.64** | 0.10 |
| Wastewater |  | **0.00** | **0.93** | 0.00 |  | **0.00** | **0.98** | 0.03 |
| Soil |  | 0.18 | 0.06 | 0.05 |  | 0.08 | 0.11 | 0.03 |
|  |  |  |  |  |  |  |  |  |
| **Dairies** |  |  |  |  |  |  |  |  |
| Feces from low producing cows |  | **0.02** | **0.30** | 0.02 |  | **0.00** | **0.49** | 0.03 |
| Feces from high producing cows |  | **0.00** | **0.63** | 0.01 |  | **0.00** | **0.84** | 0.04 |
| Wastewater |  | **0.03** | **0.75** | 0.03 |  | **0.00** | **0.99** | 0.00 |
| Soil |  | 0.09 | 0.24 | 0.00 |  | 0.00 | 0.31 | 0.02 |

^1^ Bold values indicates a statistically significant difference (*P* < 0.05) and high R values (> 0.25).

^2^ *P* = probability values for ANOSIM comparisons. Statistical significant separation of CONV and RWA resistome with a *P* value < 0.05

^3^ R = magnitude of difference in resistome composition between CONV and RWA samples (0 = total similarity, 1 = total dissimilarity).

^4^ Stress of ordination plot (the lower the stress, the better the representation of the data in two dimensions). Stress values < 0.1 indicates a good fit
