# Supplementary material for: Characterization of the Microbial Resistome in Conventional and “Raised Without Antibiotics” Beef and Dairy Production Systems
Source: Front Microbiol. 2019 Sep 4;10:1980. doi: 10.3389/fmicb.2019.01980 (PMC6736999; doi:10.3389/fmicb.2019.01980)
Supplement: Supplementary file 7 [file Table_7.DOCX]

Supplementary Table 7. Distribution of groups of resistance detected in samples collected from the conventional feedlot or dairy farm, but not detected in samples collected from farms without antibiotic use.

| Resistance | |  | Number of positive samples | | |
| --- | --- | --- | --- | --- | --- |
| Group | Conferring resistance to: |  | Feces^1^ | Wastewater^2^ | Soil^3^ |
| ANT4-PRIME | Aminoglycosides |  | 1 | 0 | 0 |
| APH6-PRIME | Aminoglycosides |  | 2 | 0 | 0 |
| PME | Beta-lactams |  | 2 | 0 | 0 |
| RAHN | Beta-lactams |  | 0 | 1 | 0 |
| CPS | Beta-lactams |  | 0 | 4 | 0 |
| LRA | Beta-lactams |  | 0 | 0 | 1 |
| BJP1 | Beta-lactams |  | 0 | 0 | 1 |
| OXA | Beta-lactams |  | 18 | 5 | 0 |
| FOSK | Fosfomycin |  | 4 | 0 | 0 |
| BRP | Glycopeptide |  | 1 | 0 | 0 |
| VANHA | Glycopeptide |  | 0 | 0 | 1 |
| MEXV | Multi-drugs |  | 0 | 0 | 1 |
| OQXA | Multi-drugs |  | 0 | 6 | 0 |
| OQXB | Multi-drugs |  | 0 | 8 | 0 |
| MEXH | Multi-drugs |  | 0 | 0 | 1 |
| MEXC | Multi-drugs |  | 0 | 0 | 1 |
| ROBA | Multi-drugs |  | 0 | 1 | 0 |
| ERM | MLS^4^ |  | 8 | 7 | 2 |
| ERMS | MLS |  | 0 | 0 | 1 |
| ERMT | MLS |  | 9 | 0 | 0 |
| OLEI | MLS |  | 0 | 0 | 1 |
| OLEB | MLS |  | 0 | 0 | 2 |
| CARA | MLS |  | 0 | 0 | 3 |
| LNUF | MLS |  | 3 | 13 | 0 |
| SULI | Sulfonamides |  | 6 | 1 | 0 |
| TET31 | Tetracyclines |  | 1 | 1 | 0 |
| TETG | Tetracyclines |  | 2 | 0 | 0 |
| TETS | Tetracyclines |  | 1 | 0 | 0 |
| DFRF | Trimethoprim |  | 2 | 1 | 0 |

^1^ Total of 32 fecal samples, ^2^ Total of 16 wastewater samples, ^3^ Total of 16 soil samples

^4^ Macrolide-Lincosamide-Streptogramin
