# Supplementary material for: Characterization of the Microbial Resistome in Conventional and “Raised Without Antibiotics” Beef and Dairy Production Systems
Source: Front Microbiol. 2019 Sep 4;10:1980. doi: 10.3389/fmicb.2019.01980 (PMC6736999; doi:10.3389/fmicb.2019.01980)
Supplement: Supplementary file 8 [file Table_8.DOCX]

Supplementary Table 8. Ten most abundant^1^ unique groups of resistance detected in the different types of samples (e.g., CFX was detected in feces but it was not detected in wastewater and soil samples).

| **Unique for feces** | |  | **Unique for wastewater** | |  | **Unique for soil** | |
| --- | --- | --- | --- | --- | --- | --- | --- |
| Group | Class |  | Group | Class |  | Group | Class |
| CFX | Beta-lactam |  | TET39 | Tetracycline |  | CEOB | Multi-drug |
| MDTC | Multi-drug |  | OQXB | Multi-drug |  | RPH | Rifampin |
| ACRF | Aminoglycoside |  | MEXT | Multi-drug |  | VANRO | Glycopeptide |
| PMRC | CAP^2^ |  | OQXA | Multi-drug |  | NOVA | Aminocoumarin |
| EVGS | Multi-drug |  | CPS | Beta-lactams |  | TLRC | MLS^3^ |
| MDTB | Multi-drug |  | EREA | MLS^3^ |  | MEXB | Multi-drug |
| ACRD | Aminoglycoside |  | ROBA | Multi-drug |  | DRRA | Multi-drug |
| MDTO | Multi-drug |  | RAHN | Beta-lactam |  | MEXQ | Multi-drug |
| MDTP | Multi-drug |  |  |  |  | CEOA | Multi-drug |
| PBP2 | Beta-lactam |  |  |  |  | MEXN | Multi-drug |

^1^ Based on the amount of normalized reads aligned to each group of resistance. Groups ordered from more to less abundant within each type of sample.

^2^CAP: cationic antimicrobial proteins

^3^ MLS: macrolide-lincosamide-streptogramin
