# Supplementary material for: Characterization of the Microbial Resistome in Conventional and “Raised Without Antibiotics” Beef and Dairy Production Systems
Source: Front Microbiol. 2019 Sep 4;10:1980. doi: 10.3389/fmicb.2019.01980 (PMC6736999; doi:10.3389/fmicb.2019.01980)
Supplement: Supplementary file 9 [file Table_9.DOCX]

Supplementary Table 9. Procrustes statistics to determine whether antibiotic resistance genes and microbial communities were correlated by superimposing the resistome (mechanism) and microbiome (phylum) ordination plots.

|  | Number of samples | |  | Parameters | | |
| --- | --- | --- | --- | --- | --- | --- |
|  | CONV | RWA |  | r^1^ | m2^2^ | *P*^3^ |
| **Feedlots** |  |  |  |  |  |  |
| Feces from early on feeding pens | 8 | 8 |  | 0.37 | 0.86 | 0.80 |
| Feces from late on feeding pens | 8 | 8 |  | 0.59 | 0.65 | 0.06 |
| Wastewater | 8 | 8 |  | 0.38 | 0.86 | 0.21 |
| Soil | 8 | 8 |  | 0.40 | 0.84 | 0.37 |
|  |  |  |  |  |  |  |
| **Dairies** |  |  |  |  |  |  |
| Feces from low producing cows | 8 | 8 |  | 0.34 | 0.88 | 0.48 |
| Feces from high producing cows | 8 | 8 |  | 0.37 | 0.86 | 0.60 |
| Wastewater | 8 | 8 |  | 0.44 | 0.81 | 0.11 |
| Soil | 8 | 8 |  | 0.47 | 0.77 | 0.13 |

^1^ r = coefficient of correlation between resistome and microbiome ordinations;

^2^m^2^: residual sum of squares after contrasting resistome and microbiome ordinations (higher m^2^ values indicate more dissimilarity between the two ordinations).

^3^*P* = probability values for each procrustes comparison. Statistical significant correlation between resistome and microbiome ordinations with a *P* value < 0.05
