# Supplementary material for: Characterization of the Microbial Resistome in Conventional and “Raised Without Antibiotics” Beef and Dairy Production Systems
Source: Front Microbiol. 2019 Sep 4;10:1980. doi: 10.3389/fmicb.2019.01980 (PMC6736999; doi:10.3389/fmicb.2019.01980)
Supplement: Supplementary file 11 [file Table_11.DOCX]

Supplementary Table 11. Resistome and microbiome richness (R) and Shannon’s diversity (D).

|  |  | **Resistome level** | | | | | | | |  | **Microbiome** | |
| --- | --- | --- | --- | --- | --- | --- | --- | --- | --- | --- | --- | --- |
|  |  | Class | |  | Mechanism | |  | Group | |  | Phylum | |
| **Factors and levels** |  | R^1^ | D^2^ |  | R | D |  | R | D |  | R | D |
| *Production practice* |  |  |  |  |  |  |  |  |  |  |  |  |
| Conventional |  | 7 | 1.1 |  | 15 | 1.4 |  | 45 | 2.5 |  | 35 | 1.2 |
| Without antibiotics |  | 6 | 1.0 |  | 12 | 1.2 |  | 34 | 2.3 |  | 36 | 1.3 |
| Significance^3^ |  | ns | ns |  | * | * |  | * | ns |  | ns | ns |
|  |  |  |  |  |  |  |  |  |  |  |  |  |
| *Type of sample* |  |  |  |  |  |  |  |  |  |  |  |  |
| Feces, early on feed pens^4^ |  | 7 | 0.8 |  | 19 | 1.1 |  | 63 | 2.4 |  | 36 | 1.6 |
| Feces, late on feed pens^4^ |  | 7 | 0.8 |  | 20 | 1.1 |  | 67 | 2.4 |  | 36 | 1.5 |
| Feces, low producing cows^5^ |  | 7 | 1.4 |  | 15 | 1.7 |  | 43 | 2.6 |  | 35 | 1.4 |
| Feces, high producing cows^5^ |  | 7 | 1.4 |  | 17 | 1.7 |  | 59 | 2.3 |  | 36 | 1.5 |
| Wastewater |  | 4 | 0.8 |  | 9 | 1.2 |  | 20 | 2.0 |  | 35 | 1.0 |
| Soil |  | 7 | 1.2 |  | 12 | 1.3 |  | 21 | 2.3 |  | 36 | 0.9 |
| Significance |  | * | * |  | * | * |  | * | * |  | ns | * |
|  |  |  |  |  |  |  |  |  |  |  |  |  |
| *Type of cattle* |  |  |  |  |  |  |  |  |  |  |  |  |
| Dairy |  | 6 | 1.1 |  | 12 | 1.4 |  | 32 | 2.3 |  | 36 | 1.2 |
| Feedlot |  | 7 | 0.9 |  | 16 | 1.2 |  | 46 | 2.5 |  | 36 | 1.2 |
| Significance |  | * | * |  | * | ns |  | * | ns |  | ns | ns |

^1^R (richness): number of different antibiotic resistance features in the resistome (class, mechanism and group level) and number of different phyla in the microbiome

^2^D (Shannon’s diversity): index that accounts for both abundance and evenness of the antibiotic resistance features or taxa present in the resistome and microbiome, respectively.

^3^*P*-value < 0.05: * (significant); *P*-value > 0.05: ns (not significant)

^4^ Feces collected from beef cattle in feedlots

^5^ Feces collected from milking cows in dairy farms
